# The Intergenerational Impacts of Paternal Diet on DNA Methylation and Offspring Phenotypes in Sheep

**DOI:** 10.3389/fgene.2020.597943

**Published:** 2020-11-05

**Authors:** Nicole Gross, Todd Taylor, Thomas Crenshaw, Hasan Khatib

**Affiliations:** Department of Animal and Dairy Sciences, University of Wisconsin-Madison, Madison, WI, United States

**Keywords:** nutritional epigenetics, DNA methylation, non-genomic inheritance, paternal diet, rumen-protected methionine

## Abstract

Knowledge of non-genomic inheritance of traits is currently limited. Although it is well established that maternal diet influences offspring inheritance of traits through DNA methylation, studies on the impact of prepubertal paternal diet on DNA methylation are rare. This study aimed to evaluate the impact of prepubertal diet in Polypay rams on complex traits, DNA methylation, and transmission of traits to offspring. A total of 10 littermate pairs of F0 rams were divided so that one ram was fed a control diet, and the other was fed the control diet with supplemental methionine. Diet was associated with earlier age at puberty in treatment vs. control F0 rams. F0 treatment rams tended to show decreased pubertal weight compared to control rams; however, no differences were detected in overall growth. A total of ten F0 rams were bred, and the entire F1 generation was fed a control diet. Diet of F0 rams had a significant association with scrotal circumference (SC) and weight at puberty of F1 offspring. The paternal diet was not significantly associated with F1 ram growth or age at puberty. The DNA methylation of F0 ram sperm was assessed, and genes related to both sexual development (e.g., *DAZAP1*, *CHD7*, *TAB1*, *MTMR2*, *CELSR1*, *MGAT1*) and body weight (e.g., *DUOX2*, *DUOXA2*) were prevalent in the data. These results provide novel information about the mechanisms through which the prepubertal paternal diet may alter body weight at puberty and sexual development.

## Introduction

Genomic prediction is a pillar of animal breeding in the livestock industry. However, some traits are more challenging to predict due to their complexity. For instance, livestock health traits have been shown to have as low as 20% heritability (Bastin et al., 2016), and reproductive traits have been shown to have less than 5% heritability (Berglund, 2008; Liu et al., 2008). In recent studies, the inclusion of biological information and functional annotation data in genomic prediction models has been shown to improve the prediction of dairy bull fertility (Abdollahi-Arpanahi et al., 2017; Nani et al., 2019). Thus, gaining a better understanding regulation of gene function and non-genomic inheritance is increasingly important and may serve as an avenue to explain variation in lowly-heritable traits.

Maternal nutrition impacts the inheritance of traits, which are challenging to predict using only genomic data. Genomic data explain only 6% of heritability for type 2 diabetes (Zeggini et al., 2008) and only 5.2% of heritability for high-density lipoprotein (HDL) cholesterol (Kathiresan et al., 2008 as reviewed by Manolio et al., 2009). Several groundbreaking studies on the Dutch famine (Roseboom et al., 2001) revealed that maternal undernutrition during gestation is associated with altered glucose tolerance (Ravelli et al., 1998) and incidence of coronary heart disease (Roseboom et al., 2000) for children of affected mothers.

Epigenetics, which describes changes in gene expression that do not alter the DNA nucleotide sequence (Feil, 2006), offers a possible explanation for non-genomic inheritance. DNA methylation is one of the most heavily studied epigenetic marks (Reik and Dean, 2001). This mark is created when a methyl group is added to the fifth position of a cytosine ring (Pinney, 2014). DNA methylation is most commonly studied in the context of cytosine-guanine (CpG) dinucleotides, but methylation can also occur at non-CpG sites (Pinney, 2014). The addition of these methyl groups to the DNA sequence can affect the transcription of genes by interfering with the binding of transcription factors (Comb and Goodman, 1990) or by recruiting DNA binding factors that can alter chromatin activity (Lewis et al., 1992). DNA methylation occurs as an output of one-carbon metabolism (OCM), the metabolic pathway for the utilization of dietary methyl donors via folate transfer of methyl groups from methionine (Ikeda et al., 2012). Therefore, DNA methylation has become a heavily studied epigenetic mark for nutritionally-focused studies that evaluate the transgenerational epigenetic inheritance of complex traits.

A breakthrough study on the agouti gene showed that supplementing pregnant female mice with genistein, an isoflavone commonly found in soy products, resulted in a darkened coat color and decreased obesity in offspring (Dolinoy et al., 2006). The authors also found that the offspring of these mothers had increased DNA methylation at six CpG sites in a retrotransposon upstream of the agouti gene (Dolinoy et al., 2006). These effects were recapitulated by supplementing other methyl donors to pregnant mice (Cropley et al., 2006). Following the agouti studies, our research demonstrated that the supplementation of methionine to cows at the preimplantation stage of embryo development significantly altered the expression of 276 genes in blastocysts, decreasing gene expression in most cases (Peñagaricano et al., 2013). In another study, we demonstrated that pregnant sheep fed diets with different methyl donor content (hay vs. corn) during late gestation led to alterations of fetal programming marked by DNA methylation in muscle and fat tissues (Lan et al., 2013; Peñagaricano et al., 2014; Namous et al., 2018). The majority of the research on the dietary influence of transgenerational inheritance has been focused on the maternal diet during pregnancy. However, relatively few studies have assessed whether exposure of males to altered diets can impact germline transmission of DNA methylation to offspring.

In particular, the prepubertal period is a sparsely studied window of susceptibility to *de novo* DNA methylation of genes (Soubry et al., 2014). Nevertheless, there is epidemiological evidence that during this developmental phase, the diets of males can impact complex traits in their offspring. Using data from three cohorts born in 1890, 1905, and 1920 from the Överkalix parish in northern Sweden, Kaati et al. (2002) showed that low food availability resulting in a suppressed growth of the father before puberty led to decreased cardiovascular disease mortality in the next generation. Further, if the paternal grandfathers were exposed to excess food during the prepubertal growth period, their grandchildren had higher mortality from diabetes (Kaati et al., 2002).

Additionally, the transgenerational epigenetic inheritance of imprinted genes in which the paternal DNA methylation is transmitted to the offspring is well established (Elhamamsy, 2017). Imprinted genes rely on correct patterns of paternal germline DNA methylation to allow for mono-allelic inheritance of expression of the gene from either the maternal or paternal locus (Elhamamsy, 2017). The disruption of male or female germline DNA methylation can, therefore, lead to various imprinting disorders (Elhamamsy, 2017). The prepubertal time window is a possible point for *de novo* methylation of imprinted genes (Lucifero et al., 2002; Soubry et al., 2014). So further evaluation of the prepubertal paternal diet on male sperm DNA methylation and its transfer to offspring will add critical information to the non-genomic inheritance of traits in mammals.

Previously, a mammalian model for obesity was designed to evaluate whether the prepubertal paternal diet impacted sperm DNA methylation in both the treated males and their offspring (Carone et al., 2010). In the study, researchers fed high-fat diets compared to control and low protein diets to mice from weaning until puberty. The group found that paternal diets affected cholesterol and lipid metabolism in offspring (Carone et al., 2010). The offspring had a modest difference in DNA methylation of the peroxisome proliferator activated receptor alpha (*PPAR*α) in their livers, but sperm DNA methylation of the gene was not altered (Carone et al., 2010). Although alternative epigenetic mechanisms such as sperm RNAs and histone modifications can affect offspring RNA expression (Shi et al., 2016), the ability of DNA methylation to alter the presence of such molecules should not yet be discounted (Comb and Goodman, 1990; Lewis et al., 1992). To our knowledge, no other mammalian studies evaluating the impact of the prepubertal male diet on DNA methylation transmission have been conducted. Yet, the ability of the paternal germline to transmit environmentally-induced DNA methylation marks has been questioned (Baxter and Drake, 2019). The timing of pubertal development between animal models can differ substantially. In mice, sexual maturation closely follows fetal and neonatal development, and it is more challenging to pinpoint the initiation of pubertal development. However, both sheep and primates have a distinct prepubertal period, with a far longer duration than the prepubertal period in mice (Plant, 2015). Therefore, it is imperative to perform studies using additional species and dietary conditions to understand male germline transmission of DNA methylation better.

Further, there is still a need to understand the manifestation of complex traits related to the paternal diet. The primary focus of this study was to assess the intergenerational impact of paternal diet on DNA methylation and offspring phenotypes. Diets differing in a single nutrient were fed to littermate pairs of Polypay rams from weaning to puberty. The impacts of the diets on traits in F0 rams were evaluated. Additionally, alterations in sperm DNA methylation of the F0 germline and transfer of traits from the F0 to the F1 generation were studied.

## Materials and Methods

### Ethics Statement

Experimental protocols were approved by the Animal Care and Use Committee at the University of Wisconsin-Madison (ID: A005171-R01).

### Supplementation of Rumen-Protected Methionine (RPM) in Diets of F0 Rams

In total, 10 littermate pairs of Polypay rams were split so that one animal from each pair was assigned the treatment diet, and the other animal was assigned the control diet at random. The control diet was a general basal diet (see Supplementary Table S1), and the treatment was the same control diet plus an additional top-dress (0.22% addition) of methionine supplied in an encapsulated form to escape rumen degradation (RPM Smartamine^®^, Adisseo, Alpharetta, GA, United States). Rams were separated into individual pens and fed 0.68 kg of the control or methionine supplement twice daily from 10.7 to 13.6 weeks of age until puberty. Treatment animals received a top-dress supplement of 1.5 g of the rumen-protected methionine (RPM) product at each feeding. Between feedings, rams were group-housed and had free access to forage (a mixture of orchard grass and alfalfa hay) and water. After puberty, all rams were allowed *ad libitum* access to the control diet.

### Semen Evaluation

Semen was collected weekly via electroejaculation technique, using the Lane Pulsator IV (Lane Manufacturing Inc., Denver, CO, United States). There was an average of 7.3 days between collections. Before each collection, ram body weight was recorded, and scrotal circumference (SC) was measured at the widest point in the scrotum using a flexible measuring tape. For semen collection, rams were restrained, and the ventrally oriented three-electrode probe was inserted into the rectum. Pulses were performed using the pre-programmed mode and maintained until the Lane Pulsator IV reached step 6, ending before step 6 if rams ejaculated earlier. Ejaculates were collected into a graduated conical vial. A 30 μL portion of raw semen was removed and added to a 0.1% paraformaldehyde (PFA) solution at a 1:2 dilution. The remaining semen was transported to the laboratory in a pre-warmed (37°C) egg yolk-based semen extender. Semen concentration was evaluated using the PFA-preserved sample, with a hemocytometer. Then, total sperm per ejaculate was further calculated by multiplying sperm concentration of the ejaculate by the total volume. The motility of semen transported in egg yolk extender was evaluated using computer-assisted sperm analysis (CASA) with a Hamilton Thorne semen analyzer (Hamilton-Thorne Research, Beverly, MA, United States) (Hoflack et al., 2007). To ensure rams were pubertal before treatment diet removal, puberty was stringently defined as two qualifying samples from subsequent weeks. A qualifying collection of semen has at least 50 × 10^6^ sperm per ejaculate and ≥10% motility (Mukasa-Mugerwa and Ezaz, 1992). The remaining semen from each qualifying sample was washed with phosphate-buffered saline, pelleted by centrifugation, and stored in RNAlater at −80°C.

Semen stored in PFA was washed twice in distilled water, and then a smear was created on a glass slide and dried rapidly at 39°C using a heated slide warmer. Sperm were examined under a phase-contrast microscope with a 40x objective. Two observers were used to evaluate sperm morphology, and a minimum of 200 sperm per animal was assessed. Total percent abnormal spermatozoa, primary defects (e.g., short head, detached head, long head, pyriform, or knobbed acrosomes), and secondary defects (e.g., bent/broken tail, midpiece abnormalities, translocating droplets) were measured. The average from the two observers was used in the final analysis.

### F0 Generation Statistical Analysis

All F0 data were assessed using a linear mixed model. The diet was included as a fixed effect, and the ram pair was included as a random effect. Dependent variables that were assessed individually were the age at puberty, weight at puberty, SC at puberty, average daily gain (ADG) from the beginning of the trial until puberty, percent abnormal spermatozoa, primary sperm defects, and secondary sperm defects. Additionally, weight was collected each week, and SC was recorded until puberty. For weight traits, the stepwise forward selection was performed using a *p*-value cutoff of 0.2 to evaluate whether or not birth weight should be included as a fixed effect in the mixed model. Through forward stepwise selection, the birth weight did not account for a significant source of variation and was not included in the final model.

### Breeding F0 Generation Rams

The ewes used in breeding were synchronized using a vasectomized ram. A total of 10 animals (pair 2, pair 3, pair 5, pair 6, and pair 10) were chosen for breeding. The most phenotypically extreme pairs of rams were chosen, based on the results observed for both age and weight at puberty. Within this subset of bred animals, the treatment group had 2.8 weeks earlier age at puberty. The selection of extreme animals was performed under the assumption that an earlier age at puberty was induced by the treatment, due to eight of 10 pairs of rams showing earlier age at puberty. Through this assumption, pair 9 demonstrated abnormal results compared to the average and was assumed to represent a pair less susceptible to the effects of the treatment. Therefore, pair 9 was not used in breeding. Selected rams were penned individually with a group of eight to nine Polypay ewes each for two breeding cycles. After the first cycle, an additional ejaculate was collected and stored as described above. A marking harness was used to ensure the breeding of each female. Fetuses were scanned and counted via ultrasound at 8.4–10.6 weeks of pregnancy.

### F1 Generation Management and Trait Evaluation

An average of 8.3 litters and 19.4 offspring were produced from each F0 ram. Overall, averages of several F1 traits were taken for comparison of treatment vs. control. For each F0 ram, the percent fetuses resorbed out of the total number of lambs born, percent mortality of all F1 offspring up to 48 h, the overall sex ratio of F1 offspring, sex ratio per litter, total offspring per litter, and the average of F1 birth weights were calculated. Offspring birth weight averages were assessed both as an overall average and by F1 sex. Lamb litters with greater than two animals per birth had any additional lambs removed after 48 h of colostrum consumption, so that a maximum of two lambs were raised naturally per ewe. When possible, excess ram lambs born from litters with greater than two lambs were fostered to another ewe. Weaning of naturally reared and fostered rams occurred at an average of 89 days of age. The remaining rams and ewes raised as a group on an artificial milk replacer were weaned at an average of 39 days of age. Ewes were preferentially selected to be artificially reared when possible. After weaning, all animals were fed the basal control diet for the remainder of the trial. A linear mixed model was used for statistical analysis. F0 diet and litter of F0 rams were included as a fixed effect.

After all animals were weaned, rams were randomly mixed and separated into three pens. Semen collections and puberty evaluations were performed as described above for F0 rams. There was an average of 6.9 days between collections. A total of 62 rams (35 treatment and 27 control offspring) produced two qualifying samples of semen from subsequent weeks and were included in the final analysis of age at puberty, SC at puberty, and weight at puberty.

In total, 15 rams (six control and nine treatment offspring) were removed from puberty trait analysis at the end of the trial, as they did not produce a qualifying ejaculate by 7 months of age. Since these animals were weighed at weekly intervals until the end of the trial, they were included in the growth analysis. The R package growthcurver was used to evaluate the growth of 77 rams (44 treatment and 33 control offspring). The growthcurver package calculates the inflection point of growth, growth rate, and carrying capacity (the point at which growth plateaus) using the non-linear least-squares Levenberg–Marquardt algorithm (Fan, 2012; Sprouffske and Wagner, 2016). A total of 20 time-points from birth until rams reached an average of 211 days of age were utilized for the growth analysis.

All F1 data were evaluated using linear mixed models. The diet was included as a fixed effect, and the F0 ram pair and the F1 pen were included as random effects. Dependent variables analyzed were weight at puberty, age at puberty, SC at puberty, the inflection point of the growth curve, growth rate, and carrying capacity. Rams were classified into different rear types as singly raised, artificially reared, naturally reared pairs, and fostered. A forward stepwise selection was used to determine the appropriate model for each dependent variable. The rear type was evaluated as a potential fixed effect for the final statistical model, using a *p*-value cutoff of 0.2. The rear type was included as a fixed effect for analyses of weight at puberty, SC at puberty, the inflection point of the growth curve, and carrying capacity.

### F0 Semen DNA Methylation Evaluation

Semen DNA was extracted using the phenol-chloroform method. DNA quality and quantity were assessed via nanodrop and gel electrophoresis. Reduced representation bisulfite sequencing (RRBS) using *Msp*I-digested DNA was performed at the University of Illinois, Urbana-Champagne. Quality control of raw reads was carried out with FastQC software (version 0.11.8, Babraham Bioinformatics, United Kingdom). TrimGalore (version 0.5.0, Babraham Bioinformatics, United Kingdom) was used on default mode to filter data (without the RRBS setting per NuGen recommendations), so that reads of 20 nucleotides or longer were retained. Then, data were passed through NuGen’s diversity trimming script to remove any reads that do not contain a *Msp*I site signature YGG at the 5’ end. After trimming, reads were aligned to the sheep genome Oar_v3.1 with Bismark (version 0.20.1, Babraham Bioinformatics, United Kingdom). Next, the sequencing data were deduplicated using a NuGen-provided script. The Bismark methylation extractor was used to extract methylation calls at a single-base resolution (Krueger and Andrews, 2011). The cutoff for a minimum number of reads was set to five. Calculations of read counts and methylation levels were performed using the methylKit package (version 1.8.1) (Akalin et al., 2012). A minimum of 10 reads per cytosine was used as a cutoff for further analysis. Both differentially methylated cytosines (DMCs) and differentially methylated regions (DMRs) were evaluated, using a minimum difference of 25% methylation as a cutoff and a *q*-value of <0.1. The DMCs or DMRs which had higher methylation in treatment animals were considered as hypermethylated, while those with lower methylation in treatment animals were considered as hypomethylated. Annotation of genomic regions was performed using the methylKit package (version 1.8.1) (Akalin et al., 2012). Exons and introns which overlapped with DMCs and DMRs were identified, in addition to DMCs and DMRs, which were within 20 kb from transcription start sites (TSSs) of genes. Further, repetitive elements were downloaded from the University of California, Santa Cruz (UCSC) table browser data retrieval tool (Karolchik et al., 2004), and overlaps with the DMCs and DMRs were also annotated. This included DMCs, which were within the repetitive elements, and DMRs, which overlapped with the elements. Additionally, DMRs with >50% overlap were also separated for consideration. Functional enrichment analysis was performed using G:Profiler (Raudvere et al., 2019), which provides enrichment analysis of Gene Ontology (GO) terms, KEGG pathways, and transcription factors from the TRANScription FACtor database (TRANSFAC). The organism was set to *Homo sapiens* for exploratory purposes because *Ovis Aries* is not comprehensively represented in the functional databases. Further, Medical Subject Headings (MeSH) analysis was performed under *H. sapiens* using BioLitMine (v0.7.0) (Hu et al., 2020). Enrichment for MeSH terms from the Diseases (C) and Chemicals and Drugs (D) was evaluated.

## Results

### The Effects of RPM Supplementation on F0 Rams

**FIGURE 1:**
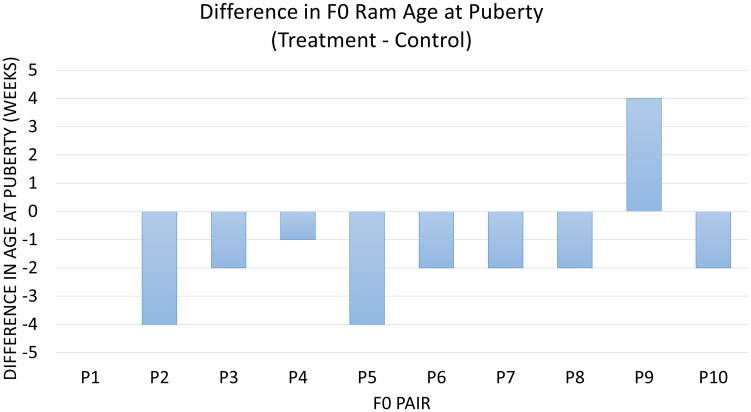
The difference in age at puberty in all littermate pairs of F0 rams. Data are represented as treatment minus control in weeks of age at puberty. Diet was associated with age at puberty (*P* = 0.03), which differed by an overall average of –1.5 weeks in treatment vs. control pairs of animals. Pair 1 has a difference of 0.

Diet was significantly associated with age at puberty between the 20 F0 treatment and control animals (*P* = 0.03). On average, treatment animals reached this milestone 1.5 weeks (9.8 days) earlier than control animals (Figure 1). The ADG of treatment and control animals until puberty was not different (*P* > 0.1). Overall, treatment animals tended to achieve puberty at a lighter weight and were an average of 2.2 kg lighter than control animals at puberty (*P* = 0.08) (Supplementary Figure S1). The animals receiving RPM also tended to have a higher percent abnormal sperm at puberty (*P* = 0.08), with 5.7% more abnormal sperm in treatment compared to control animals. There was no detectable impact of the diet on factors such as sperm motility at puberty, the volume of ejaculate at puberty, SC at puberty, and total sperm per ejaculate (*P* > 0.1).

### Intergenerational Impacts of F0 RPM Supplementation on F1 Generation Phenotypes

The number of total lambs born from treatment rams was 101, and the number born from control rams was 93. There was no significant relationship to diet for percent fetuses resorbed out of the total number of lambs born, percent mortality of all F1 offspring up to 48 h, the overall sex ratio of F1 offspring, total lambs born per litter, or the average of F1 birth weights (*P* > 0.1). The sex ratio within each litter tended to show an association with the dietary treatment of F0 rams (*P* = 0.05). On average, the treatment littermate F0 rams had 15.3% more males per litter than their control littermates. Notably, the overall average ratio of males per litter for treatment F0 litters was 55.1%, while the average ratio of males per litter for control F0 litters was 39.7%.

**FIGURE 2:**
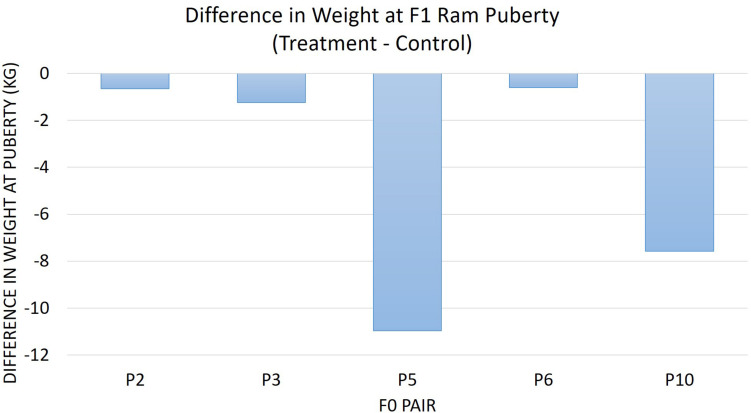
The difference in weight at F1 ram puberty is represented as treatment minus control. The average for the F1 offspring was evaluated and compared based on the F0 littermate pairs of their sire. F1 treatment rams were 4.21 kg lighter than the F1 control offspring descended from the same F0 litter pair (*P* = 0.04).

**FIGURE 3:**
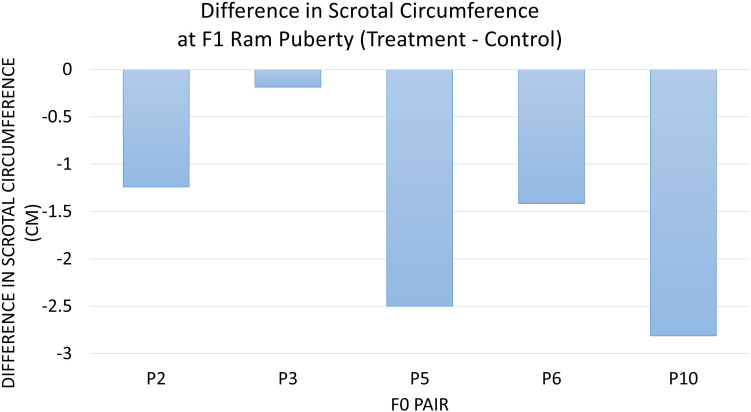
The difference in scrotal circumference at F1 ram puberty is represented as treatment minus control. The average for the F1 offspring was evaluated and compared based on the F0 littermate pairs of their sire. F1 treatment rams had 1.6 cm smaller SC than the F1 control offspring descended from the same F0 litter pair (*P* = 0.007).

The diet of F0 rams was associated with weight at puberty (*P* = 0.04) (Figure 2) and SC at puberty (*P* = 0.007) (Figure 3) for F1 offspring. On average, F1 treatment rams were 4.2 kg lighter and had 1.6 cm smaller SC at puberty than the F1 control rams. There was no substantial influence of the F0 diet on age at puberty for the F1 generation (*P* > 0.1). Additionally, sperm motility, ejaculate volume, and total sperm/ejaculate at F1 ram puberty were not noticeably affected by dietary treatment of F0 rams (*P* > 0.1). Similarly, there were no notable effects of diet on the growth of F1 rams for the measures of inflection point, growth rate, or carrying capacity of the growth curves (*P* > 0.1).

### The Identification of DMRs and DMCs in F0 Ram Sperm

**FIGURE 4:**
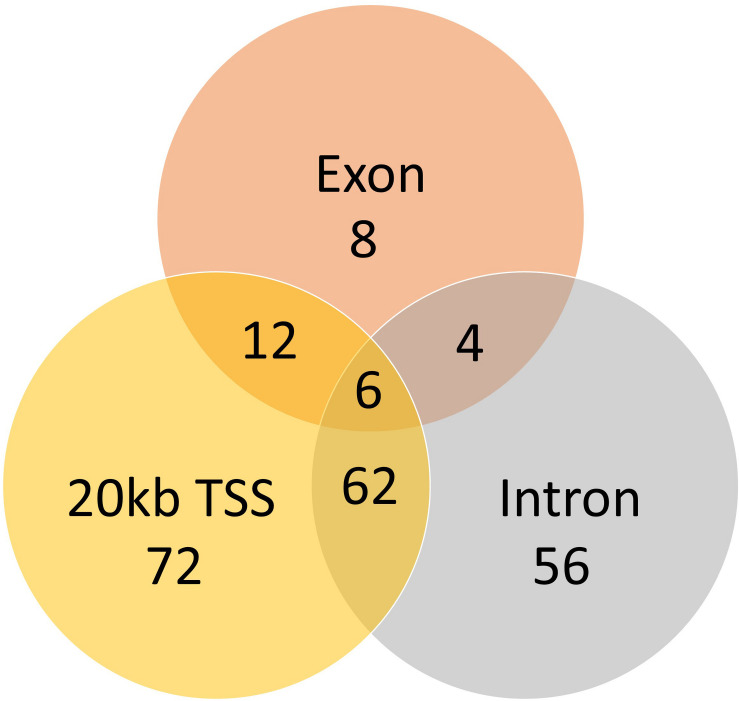
Venn diagram of the DMCs mapped to genomic regions.

**FIGURE 5:**
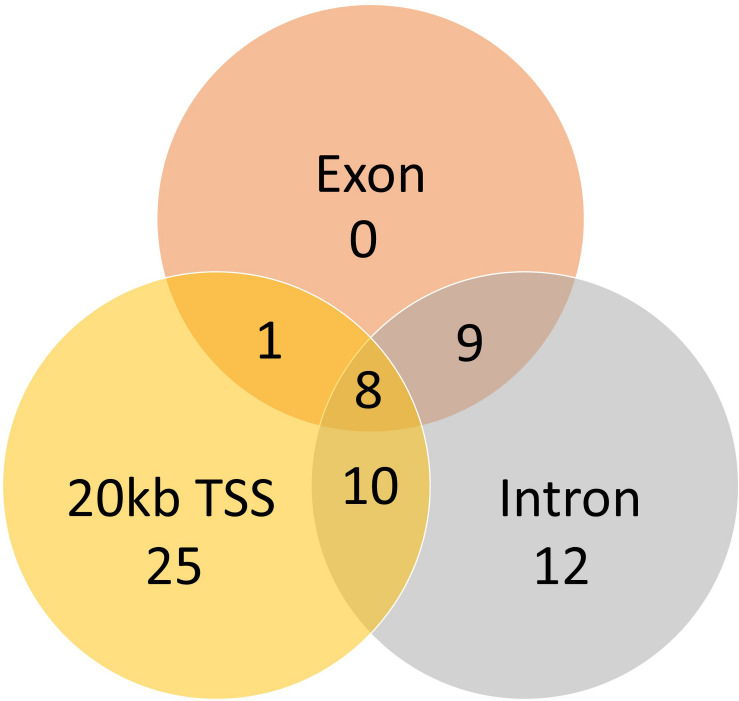
Venn diagram of the DMRs which mapped to genomic regions.

**FIGURE 6:**
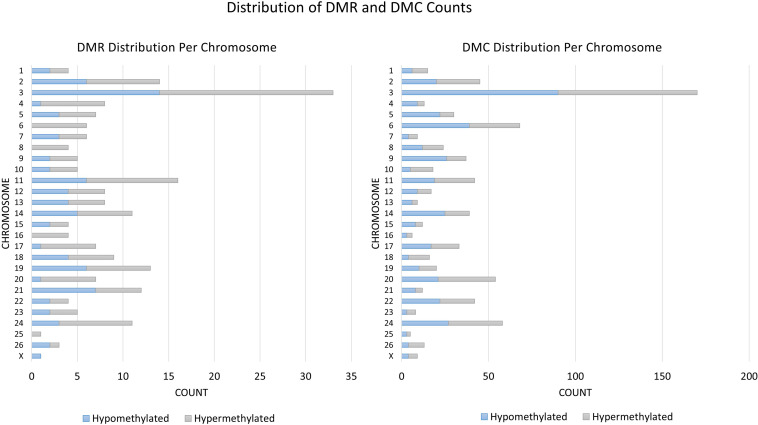
Distribution of DMRs and DMCs by chromosome.

A total of 824 DMCs (FDR < 0.1) and 216 DMRs (FDR < 0.1) were identified in the sperm of F0 rams to produce the F1 generation. Of the DMCs, 426 were hypomethylated, and 398 were hypermethylated in treatment compared to control animals. There were 83 hypomethylated and 133 hypermethylated DMRs between treatment and control animals. The DMCs were mapped to a total of 30 exons, 128 introns, and within 20 kb of 152 TSSs (Figure 4). The DMRs were mapped to 18 exons, 39 introns, and within 20 kb of 44 TSSs (Figure 5). Additionally, DMCs and DMRs were mapped to CpG islands (CpGi). The DMCs overlapped with 96 features of CpGi and 162 flanks (within 2000 bp) of CpGi. The DMRs were associated with 15 features of CpG and 32 flanks of CpGi (Supplementary Table S2). A total of 82 DMCs and 86 DMRs overlapped with repetitive elements (Supplementary Table S2). Further, 32 of the DMRs had >50% overlap with transposable elements (Supplementary Table S2). A single imprinted gene, *GRB10*, was found in the differentially methylated data. This gene had a single DMC located in a CpGi flank, within an intron, and within 20 kb of a TSS. This site was hypomethylated in treatment compared to control animals. The overall counts of DMRs and DMCs are found in Figure 6.

There were no GO terms or KEGG pathways significantly enriched in the over differentially methylated genes. However, the assessment of transcription factors revealed 49 significantly enriched terms (Supplementary Table S3). These included transcription factor AP-2, GKLF, E2F-3, SP2, SP1, PAX5, BTEB1, and CPBP, among others (Table 1). MeSH analysis revealed 514 significantly enriched terms. Some Disease category terms included were Pediatric Obesity, Genetic Predisposition to Disease, Dwarfism, and Muscle Hypotonia, Fetal Diseases, Heat Stress Disorders, Prostatic Neoplasms, and Amyotrophic Lateral Sclerosis (Table 2). The full list of terms can be found in Supplementary Table S3.

**TABLE 1:** Selected enriched MeSH terms and transcription factors^1^.

Source	Term	Number of genes	*P*-value
MeSH (Chemicals and Drugs)	RNA, Small Interfering	118	0.016497
MeSH (Chemicals and Drugs)	Membrane Proteins	120	0.001798
MeSH (Chemicals and Drugs)	Carrier Proteins	116	0.00787
MeSH (Chemicals and Drugs)	Transcription Factors	126	5.01E-15
MeSH (Chemicals and Drugs)	Recombinant Proteins	105	0.002498
MeSH (Chemicals and Drugs)	Amino Acids	47	0.029152
MeSH (Chemicals and Drugs)	Histones	46	0.000187
MeSH (Chemicals and Drugs)	Antibodies	44	0.043893
MeSH (Chemicals and Drugs)	Epidermal Growth Factor	49	0.049604
MeSH (Chemicals and Drugs)	Tumor Suppressor Protein p53	49	0.031935
MeSH (Diseases)	Amyotrophic Lateral Sclerosis	90	0.049714
MeSH (Diseases)	Prostatic Neoplasms	76	0.036599
MeSH (Diseases)	Genetic Predisposition to Disease	116	8.06E-27
MeSH (Diseases)	Pediatric Obesity	5	0.028415
MeSH (Diseases)	Dwarfism	32	0.040334
MeSH (Diseases)	Muscle Hypotonia	31	0.045181
MeSH (Diseases)	Parkinson Disease	39	0.049997
MeSH (Diseases)	Multiple Sclerosis	40	0.03529
MeSH (Diseases)	Fetal Diseases	7	0.031687
MeSH (Diseases)	Heat Stress Disorders	2	0.042298
TF	Factor: AP-2; motif: GSCCSCRGGCNRNRNN	76	4.44E-07
TF	Factor: GKLF; motif: NNCCMCRCCCN	127	5.12E-05
TF	Factor: GKLF; motif: NNCCMCRCCCN	127	5.12E-05
TF	Factor: E2F-3; motif: GGCGGGN	120	0.000123
TF	Factor: SP2; motif: GGGCGGGAC	117	0.000426
TF	Factor: CNOT3; motif: GGCCGCGSSS	61	0.000674
TF	Factor: SP1; motif: GGGGYGGGGNS	58	0.004386
TF	Factor: PAX5; motif: RNGCGTGACCNN	88	0.000954
TF	Factor: BTEB1; motif: GGGGGCGGGGCNGSGGGNGS	85	0.00183
TF	Factor: CPBP; motif: GNNRGGGHGGGGNNGGGRN	91	0.002343

*^1^MeSH = Medical Subject Headings, TF = Transcription Factors (derived from the TRANScription FACtor database).*

**TABLE 2:** Differentially methylated genes related to sexual development, fertility, and growth.

Gene symbol	Chromosome	Start	End	Methylation change	DMC/DMR	Importance	Reference(s)
*DAZAP1*	5	41228751	41229000	−40.2	DMR	Enriched in late round spermatids, before adulthood in mice; knockout of *DAZAP1* blocks the production of post-meiotic cells in spermatogenesis	Yang and Yen, 2013
*CHD7*	9	39169630	39169630	35.8	DMC	Mutations in *CHD7* are predictive of Charge Syndrome, a cause of pubertal defects in humans	Janssen et al., 2012
*TAB1*	3	214839198	214839198	40.0	DMC	Required for disruption of the blood–testis barrier and Sertoli-germ cell adhesion to allow the migration of preleptotene and leptotene spermatocytes across the barrier	Xia et al., 2006
*TAB1*	3	214840993	214840993	51.3	DMC		
*TAB1*	3	214841088	214841088	46.3	DMC		
*TAB1*	3	214830772	214830772	49.7	DMC		
*MTMR2*	15	13832021	13832021	−60.0	DMC	Knockout mice for the gene MTMR2 demonstrate progressive neuropathy and depleted spermatids and spermatocytes. Loss of MTMR2 disrupts the connections between Sertoli cells and germ cells in the seminiferous epithelium	Bolino et al., 2004
*MTMR2*	15	13832022	13832022	−54.2	DMC		
*CELSR1*	3	220867751	220868000	27.7	DMR	*CELSR1* codes for an adhesion protein that is expressed in Sertoli cells and potentially in early germ cells at the point when germ cell meiosis is initiated in mice (between postnatal days 7 and 21); mutations to *CELSR1* may lead to neural tube defects	Beall et al., 2005; Allache et al., 2012
*CELSR1*	3	220844341	220844341	−33.9	DMC		
*CELSR1*	3	220844342	220844342	−51.4	DMC		
*CELSR1*	3	220842621	220842621	−34.9	DMC		
*CELSR1*	3	220858089	220858089	35.9	DMC		
*CELSR1*	3	220881688	220881688	−26.2	DMC		
*CELSR1*	3	220882813	220882813	−43.4	DMC		
*CELSR1*	3	220891817	220891817	−41.1	DMC		
*CELSR1*	3	220858492	220858492	42.0	DMC		
*MGAT1*	5	37452239	37452239	−39.5	DMC	Knockout of *MGAT1* in spermatogonia of mice inhibits the production of sperm, causing abnormalities in cell structure at the spermatid stage and infertility; Global knockout of *MGAT1* causes complete embryonic arrest at E9.5 in mice and neural tube defects	Ioffe and Stanley, 1994; Metzler et al., 1994; Batista et al., 2012
*GRB10*	4	5228255	5228255	−34.4	DMC	Imprinted in maternal or paternal tissues, mechanism of control is unknown	Plasschaert and Bartolomei, 2015
*DUOX2* and *DUOXA2*	7	62280751	62281000	47.8	DMR	Key enzymes for thyroid hormone biosynthesis; Knockout mice show severe hypothyroidism and delayed postnatal development, including decreased weight	Grasberger et al., 2012; Weber et al., 2013

## Discussion

### Impact of Dietary Treatment on F0 Rams

The treatment animals reached puberty at an earlier age than the control animals in the F0 generation. This is particularly relevant to the livestock industry because using younger males may allow for earlier progeny testing, increase the speed of genetic selection, and reduce production costs. Although sheep are seasonal breeders, Polypay sheep can breed twice yearly (Hulet et al., 1984). Therefore, these benefits may be enhanced in this breed. However, the effects of the diet on the quality of sperm must be taken into account. Sperm morphology also tended to differ between treatment and control F0 rams. This result implies that the earlier development of mature sperm may not be favorable because the sperm produced by the treatment animals at puberty had increased abnormalities. However, this trait is confounded by the earlier age of the treatment animals because sperm quality improves as animals reach a more mature age (Fortes et al., 2012). Unfortunately, the difference observed in sperm morphology for overall treatment was absent in the rams selected for breeding. So, we were unable to conclude whether or not this impacted fertility of the rams. In future work, it would be interesting to address this question. It would also be intriguing to evaluate the sperm quality of animals fed this diet at a mature age, to understand the full impact of added RPM on sperm morphology. Of note, a single pair of rams showed an opposite change in puberty compared to the other rams in this study. In future research, it would be interesting to assess the impact of DNA methylation of males that are less susceptible to dietary influences of methionine during the prepubertal period.

Several studies have demonstrated the influence of nutrition on age at puberty. In a recent study, an increased plane of nutrition has been shown to alter the age-at-puberty (Kenny and Byrne, 2018; Kenny et al., 2018). A previous study showed that the puberty of rams could differ based on the improved nutritive value of silage (Khalifa et al., 2013). Another study on beef cattle showed that enhanced nutrition to 130% of required energy and protein was associated with approximately 1 month earlier age in puberty and enhanced sperm production, which could lead to a substantial increase in profitability [as much as Canadian Dairy Network (CDN) $2176 per collection] (Dance et al., 2016). However, this is the first study showing an effect on puberty using a single-ingredient change in methionine, a limiting amino acid in ruminant diets (Clark, 1975; Bach et al., 2000a,b). Reliable estimates of amino acid requirements, or more specifically, that of methionine, are not established for growing rams (NRC, 2007). The lack of a difference in growth traits between the treated and control groups implies that the control diet was sufficient for growth, but perhaps not for responses related to reproductive traits.

There was a tendency for F0 body weight at puberty to be associated with dietary treatment, but no association with altered SC was seen. Generally, SC is correlated with body weight (Coulter and Foote, 1977), and the inflection of SC growth has been used as a marker for ram puberty (Emsen, 2005). The causes of stable SC despite differences in pubertal body weight should be further explored in future studies.

### Intergenerational Effects of Paternal Diet on the F1 Generation

The treatment animals weighed less at puberty for both the F0 and the F1 generations and were therefore capable of achieving puberty at a lighter weight than control animals. Interestingly, there was no difference in growth for either the F0 or F1 generations. The F0 generation had an earlier age at puberty, which could explain the difference in weight of F0 rams without a change in growth. However, there was no difference in birth weight or age at puberty for the F1 generation, but the lighter weight at puberty persisted without a detectable change in growth. It is, therefore, possible that the ability to achieve puberty at a decreased weight represents an intergenerational effect of the paternal diet on the offspring. In future studies, it would be interesting to assess whether the semen quality of F1 rams would be maintained at this lower pubertal weight.

Previous studies showed that the body weight of the offspring could be influenced by a paternal diet. For instance, paternal betaine and methionine (both methyl donors) intake has been associated with decreased birth weight in humans (Pauwels et al., 2017). Additionally, a study in mice showed that reduced weight in undernourished males was also recapitulated in their normally-fed prepubertal offspring (McPherson et al., 2016). However, the age at puberty following paternal methyl donor supplementation has not been measured in previous work. Future studies should assess the mechanisms that allow puberty to occur at lighter body weight due to paternal methionine supplementation.

Interestingly, the SC of the F1 generation was significantly lower in treatment compared to control animals. However, there was not a significant difference in the SC of the F0 generation. This may indicate that decreased SC is a novel phenotype that was induced in the F1 rams. However, relatively small sample size was represented in the F0 generation. So, it is still possible that the diet induced smaller SC on some of the treated rams. For instance, seven out of 10 pairs of rams had a lower SC in treatment compared to control rams (Supplementary Figure S2). A total of two out of three F0 pairs that had higher SC in treatment vs. controls were used for breeding (P2 and P10). Yet, the average SC of F1 rams was lower in treatment compared to control offspring for all pairs. Regardless, the association of SC with paternal diet highlights the long-term implications of the prepubertal male diet.

The SC is generally positively correlated with total sperm output and subsequent testicular development (Hahn et al., 1969). Surprisingly, the F1 treatment rams were capable of equivalent sperm production at a smaller SC than F1 control rams. However, the total sperm per ejaculate at puberty was not different. It would be interesting to evaluate further whether the initial production of sperm at a lower SC leads to a difference in breeding capacity or lifetime sperm production of rams. Unfortunately, these evaluations were not possible in the timeframe of this study. Additionally, the physiological mechanisms occurring within the testis are unclear and deserve further evaluation.

Current results also indicate that the puberty differences observed in F0 rams were not transmitted to F1 rams. Weight and SC are known to be positively correlated (Coulter and Foote, 1977). Therefore, it is challenging to fully dissect the cause and effect behind alterations to these traits in F1 rams. Additionally, weight and SC have been considered factors that contribute to age at puberty (Mukasa-Mugerwa and Ezaz, 1992; Emsen, 2005; Moulla et al., 2018). So, it is challenging to determine whether the alterations in these traits stifled the intergenerational transmission of differences in age at puberty. If DNA methylation patterns or other transmissible elements that affect puberty were altered in the sperm, it is still possible that age-at-puberty could differ in the F2 generation. Indeed, it is possible for phenotypically neutral epimutations to be passed between generations (Capella and Braun, 2019). Further, these inherited epimutations can re-manifest phenotypes several generations later, when the same stimulus that caused the epimutation is applied (Duempelmann et al., 2019). In future studies, it would be intriguing to evaluate whether it is possible to re-instate the phenotype of early puberty by feeding the treatment diet to a subset of F2 rams.

### Possible Roles for DMRs and DMCs in F0 and F1 Traits

The treatment of RPM used in this study has the capability of altering DNA methylation in the sperm. Dietary methionine is used in OCM during spermatogenesis to regulate DNA methylation, maintain nucleotide synthesis, and protect DNA from damage (Singh and Jaiswal, 2012). Furthermore, DNA methylation is vital for normal spermatogenesis at puberty. For instance, when *de novo* methylation is inhibited through knockdown of DNA Methyltransferase 3 Like (DNMT3L), mice spermatocytes fail to undergo meiosis (Bourc’his and Bestor, 2004). Additionally, DNA methylation, which is altered in the male germline, can be inherited. For example, exposure of female mice to the reproductive toxicant vinclozolin during male gonadal sex determination leads to the transmission of DNA methylation through the male germline (Anway et al., 2006). The phenotypic differences of reduced spermatogenic capacity and male infertility are passed from the F1 to at least the F4 generation (Anway et al., 2005). These phenotypes were accompanied by consistently altered DNA methylation of genes in the F1 to the F4 generation (Anway et al., 2005, 2006). Therefore, DNA methylation differences observed in the F0 sperm may explain the mechanism behind the dietary effects observed in this study (Table 2).

Many F0 DMCs and DMRs are directly linked to reproductive phenotypes relevant to differences observed between treatment and control animals. The DAZ associated protein 1 (*DAZAP1*) gene had a DMR that was found within 20 kb of a TSS and overlapping with both intron and exon regions of the gene. The DMC was hypomethylated in treatment animals (−40.21%), and it has a relationship with both pubertal development and SC. *DAZAP1* codes for two transcripts which are maximally expressed at the prepubertal stage postnatal day 27. These transcripts are expressed when the testis is enriched in late round spermatids, a stage just before adulthood in mice (Yang and Yen, 2013). Although they appear and behave normally, tissue-specific knockouts of *DAZAP1* demonstrate reduced weight and testes size compared to wildtype and heterozygous littermates (Hsu et al., 2008). *DAZAP1* knockout blocks the production of post-meiotic cells in spermatogenesis. Another gene associated with puberty is chromodomain helicase DNA binding protein 7 (*CHD7*), which had a DMC within 20 kb of the TSS that was hypermethylated in treatment animals (35.81%). Mutations in *CHD7* are predictive of Charge Syndrome (Congenital Hypogonadotropic Hypogonadism), a cause of pubertal defects in humans (reviewed by Janssen et al., 2012).

Several of the genes associated with DMRs are linked to the sertoli cell function. The number of sertoli cells controls testis size and rate of sperm production (Fallah-Rad et al., 2001; Aponte et al., 2005). The biological window during which most Sertoli cells undergo proliferation is the prepubertal stage (Buzzard et al., 2000). The gene TGF-beta activated kinase 1 (MAP3K7) binding protein 1 (*TAB1*) has four DMCs, three DMCs were all 20 kb from the TSS, and were hypermethylated (40.01, 46.25, 51.26%). Another single DMC was also found in an intron, in addition to being 20 kb from the TSS of *TAB1*. The other DMC was also hypermethylated (49.71%). The *TAB1* gene produces a protein required for disruption of the blood–testis barrier and Sertoli-germ cell adhesion to allow the migration of preleptotene and leptotene spermatocytes across the barrier (Xia et al., 2006). There were two DMCs found 20 kb from the TSS and within an intron in the myotubularin related protein 2 (*MTMR2*) gene, which were both hypomethylated (−60 and −54.2%) in this study. Knockout mice for the gene *MTMR2* demonstrate progressive neuropathy and depleted spermatids and spermatocytes (Bolino et al., 2004). Additionally, loss of *MTMR2* disrupts the connections between Sertoli cells and germ cells in the seminiferous epithelium (Bolino et al., 2004). Cadherin EGF LAG seven-pass G-type receptor 1 (*CELSR1*) had a total of eight DMCs and one DMR, which overlapped both exons and introns of the gene. Two of the DMCs, which were in exons, had opposite directions of methylation (−34.86 and 42%). The DMCs within introns had five which were hypomethylated (−26.22%, −33.85%, −41.10%, −43.44%, −51.43%) and one which was hypermethylated (35.9%). The DMR was hypermethylated overall (27.69%). The DMCs in *CELSR1* were not found within the same location as the DMR, and therefore support our method of using both DMCs and DMRs to identify differential gene regulation in these samples. This gene codes for an adhesion protein that is expressed in Sertoli cells and potentially in early germ cells at the point when germ cell meiosis is initiated in mice (between postnatal days 7 and 21) (Beall et al., 2005).

A mutation in *CELSR1* may lead to neural tube defects (Allache et al., 2012). In the past, other intermediates, such as folate, that are involved in methyl transfer pathways with methionine, have been shown to serve as a remedy to prevent neural tube defects when supplemented to pregnant mothers (Wilde et al., 2014). There is some evidence that paternal methyl donor intake is critical for offspring growth and development. For instance, low paternal dietary folate in mice leads to craniofacial and musculoskeletal birth defects in offspring, in addition to altered sperm DNA methylation (Lambrot et al., 2013). Another gene in our dataset also has an established relationship with neural tube defects. Alpha-1,3-mannosyl-glycoprotein 2-beta-*N*-acetylglucosaminyltransferase (*MGAT1*) had a DMC, which was both within an exon and within 20 kb from the TSS, which was hypomethylated (−39.45%). Knockout of *MGAT1* in spermatogonia of mice inhibits the production of sperm, causing abnormalities in cell structure at the spermatid stage and infertility (Batista et al., 2012). Additionally, a global knockout of *MGAT1* causes complete embryonic arrest at E9.5 in mice (Ioffe and Stanley, 1994; Metzler et al., 1994). Intriguingly, these degenerated embryos have impairment in neural tube formation (Ioffe and Stanley, 1994; Metzler et al., 1994). In the future, paternal nutritional influence over-regulation of neural tube defects should be further investigated.

Imprinted loci and repetitive elements can both evade resetting of DNA methylation during embryonic development (Lane et al., 2003; Seisenberger et al., 2012). In this study, we identified a DMC within the imprinted gene growth factor receptor bound protein 10 (*GRB10*). Although *GRB10* is maternally imprinted in many tissues, it is paternally imprinted in some tissues, such as neuronal tissues (Plasschaert and Bartolomei, 2015). The mechanism for maternal vs. paternal control of *GRB10* imprinting is not clearly understood (Plasschaert and Bartolomei, 2015). Therefore, it is interesting to find a paternally-influenced DMC in this gene. In the future, this site should be evaluated as a possible maternal-paternal switch of the *GRB10* gene. Previously, other imprinted genes such as makorin ring finger protein 3 (*MKRN3*) and delta like non-canonical Notch ligand 1 (*DLK1*) have also been associated with the timing of puberty (reviewed by Kotler and Haig, 2018). Therefore, the *GRB10* DMC is of heightened importance and should be further evaluated in studies of placental tissues and transmission of DNA methylation in the F1 and F2 generations. A total of 82 DMCs and 86 DMRs were also found to overlap with repetitive elements. Since these regions have a higher likelihood of evading erasure during embryonic development (Lane et al., 2003), their roles should also be further explored.

Many of the differentially methylated genes are involved in pathways associated with thyroid hormone, which can affect both body weight and pubertal development. The relationship between body weight and thyroid hormone status is well established (Knudsen et al., 2005; Iwen et al., 2013; Fox et al., 2019). Further, thyroid function is a marker for both early and delayed puberty in humans (Klein et al., 2017) and sheep (Fallah-Rad et al., 2001), and is a regulator of Sertoli cell proliferation (Holsberger and Cooke, 2005). Thyroid hormone also serves as a modulator of bone acquisition during prepubertal growth (Xing et al., 2012). Therefore, it is intriguing that we have found a DMR which is hypermethylated (47.78%) and is associated with the genes coding for both of the main enzymes involved in generating the H_2_0_2_ used in thyroid hormone biosynthesis, dual oxidase 2 (*DUOX2*) and dual oxidase maturation factor 2 (*DUOXA2*) (Weber et al., 2013). The DMR covers exonic and intronic regions of *DUOXA2* and is also within 20 kb of the TSS for *DUOX2*. Inhibition of *DUOXA1* and *DUOXA2* simultaneously prevents the targeting of functional enzymes *DUOX1* and *DUOX2* to the surface of epithelial cells in order to release H_2_0_2_. Knockout mice show severe hypothyroidism and delayed postnatal development. Phenotypes of knockouts include delayed eye opening (a marker for cerebral maturation), impaired bone turnover and growth, enhanced respiratory issues, enlarged thyroid, and decreased weight (Grasberger et al., 2012). If this differential methylation is inherited and regulates gene expression of *DUOX2* and/or *DUOXA2* in offspring, it could explain the reduced weight gain of the treatment offspring compared to control offspring, which was observed in both F0 and F1 males. Additionally, epigenetic regulation of thyroid hormone-related genes may also explain earlier age at puberty in F0 animals, and has the potential to be inherited to cause decreased SC observed in F1 animals. In the future, DNA methylation differences related to thyroid hormone should be further explored to elucidate better how the paternal diet could regulate the growth of offspring.

The roles of differentially methylated genes are compelling, but a limitation of the current study was that the DNA methylation in F1 animals was not interrogated. Future studies should strive to understand the direct impact of altered DNA methylation in paternal sperm on the inheritance of DNA methylation in F1 animals. Further, it will be interesting to evaluate whether non-genomic marks associated with a minor change in the diet during the prepubertal period persist until the F2 generation. Additionally, further research should also be conducted to better understand extreme groups, such as the cohort of 15 F1 rams, which did not achieve puberty within seven months.

## Conclusion

This study is a pioneering effort for understanding the transgenerational epigenetic influence of paternal diet over complex traits. To our knowledge, it is the first study to assess the impact of a minor change to a paternal diet on offspring phenotypes. Our results provide evidence that RPM supplementation to prepubertal rams leads to altered age at puberty, with an additional tendency for the diet to impact weight at puberty. Further, we demonstrated that this diet is capable of altering DNA methylation at influential genomic regions in F0 ram gametes. The offspring of the treated rams showed differences in weight and SC at puberty, which have associations with the genes found differentially methylated in F0 ram sperm. The current results reveal possible intergenerational impacts of feeding RPM to rams and underline that understanding the far-reaching impact of the paternal diet at the prepubertal stage is critical.

## Data Availability Statement

The raw data for RRBS is found within the SRA database under SRA accession PRJNA648783.

## Ethics Statement

The animal study was reviewed and approved by the Animal Care and Use Committee at the University of Wisconsin-Madison.

## Author Contributions

NG performed the experiments, analyzed the data, and wrote the manuscript. TT managed the sheep flock and performed the breedings. TC planned the nutrient calculations and contributed to the final version of the manuscript. HK conceived the original idea and supervised the project. All authors contributed to the article and approved the submitted version.

## Conflict of Interest

The authors declare that the research was conducted in the absence of any commercial or financial relationships that could be construed as a potential conflict of interest.
