# Supplementary figures and images for: The Intergenerational Impacts of Paternal Diet on DNA Methylation and Offspring Phenotypes in Sheep
Source: Front Genet. 2020 Nov 5;11:597943. doi: 10.3389/fgene.2020.597943 (PMC7674940; doi:10.3389/fgene.2020.597943)

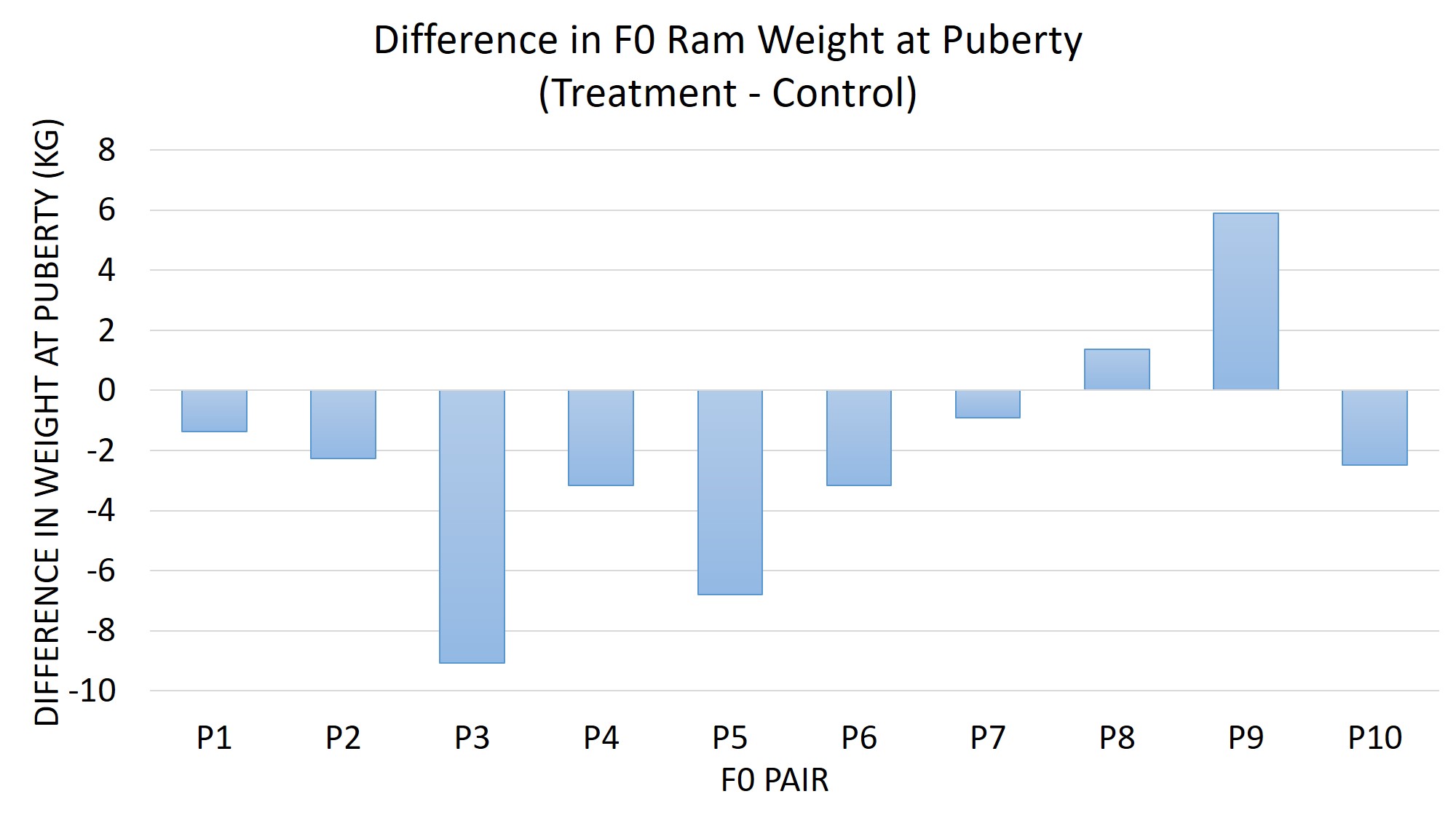

Supplement: Supplementary file 1 [file Image_1.JPEG]

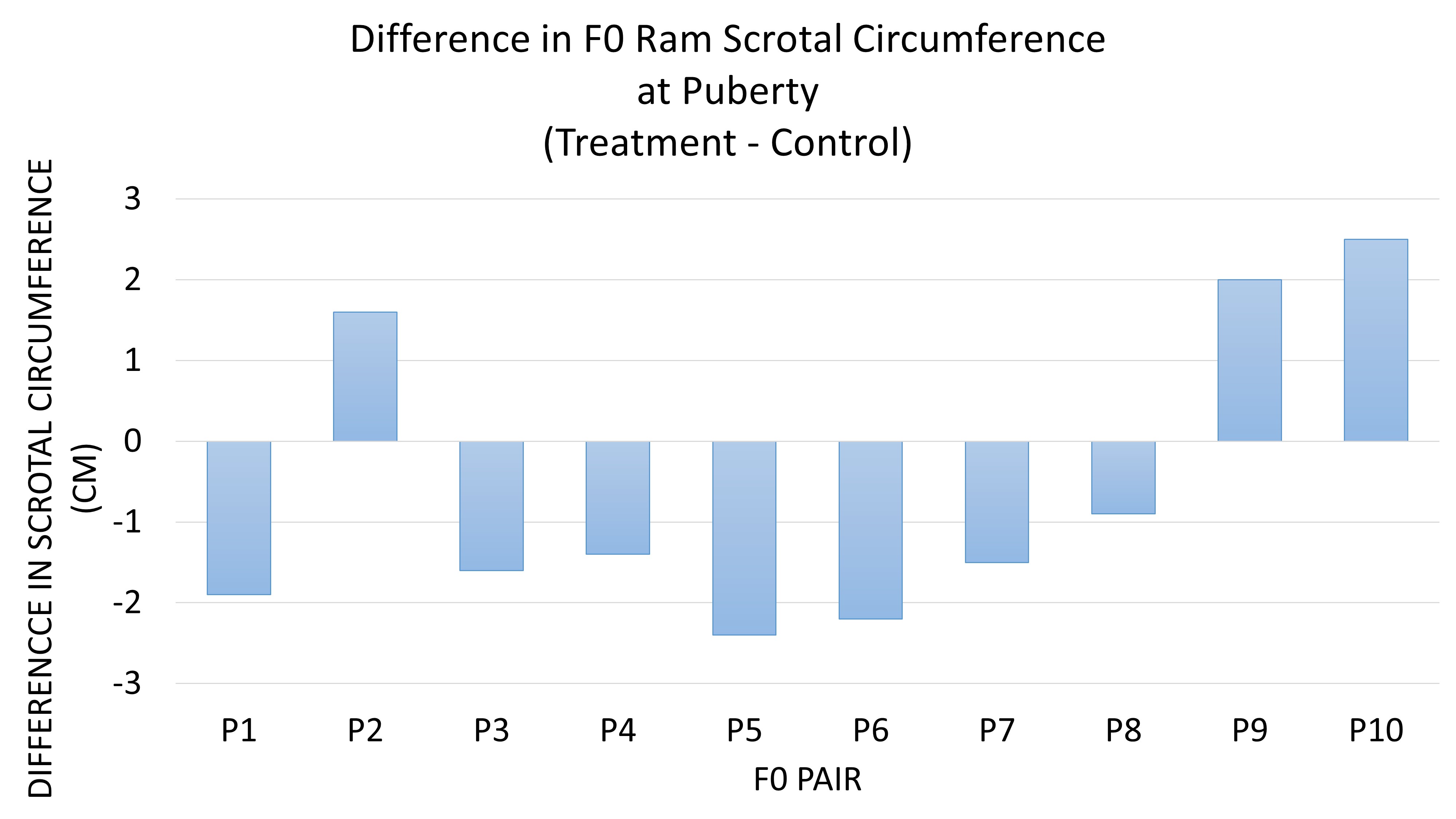

Supplement: Supplementary file 2 [file Image_2.JPEG]
