# Supplementary material for: The Intergenerational Impacts of Paternal Diet on DNA Methylation and Offspring Phenotypes in Sheep
Source: Front Genet. 2020 Nov 5;11:597943. doi: 10.3389/fgene.2020.597943 (PMC7674940; doi:10.3389/fgene.2020.597943)
Supplement: Supplementary file 3 [file Table_1.DOCX]

**Supplemental Table S1. Ingredient composition of nutrient supplement fed to rams during the growth phase ^a b^**

| Ingredients | Amount, % |
| --- | --- |
| Corn, whole grain | 67.50 |
| Lamb supplement ^c^ | 13.75 |
| Oats, whole grain with hulls | 12.50 |
| Molasses, liquid | 5.00 |
| Ammonium chloride | 0.50 |
| Calcium carbonate | 0.75 |
| Total | 100.00 |

a. Control rams were individually fed 0.68 kg of the nutrient supplement for each of 2 daily feeding periods in addition to free access to orchard grass, alfalfa hay forage. The nutrient supplement provided (dry matter basis): 13.9% crude protein, 0.83% calcium, 0.29% phosphorus and 0.26% methionine (1.51% methionine + cysteine).

b. Methionine supplemented rams were fed the same as control rams with the addition of methionine supplied as a top-dress using a rumen-stable, abomasum dispersible encapsulated methionine product (RPM, Smartamine®, Adisseo, Alpharetta, GA, USA). The top-dress addition was added as 0.22% of the nutrient supplement (1.5 g RPM for each feeding period.

c. A closed-formula, commercial lamb supplement (Lamb Grower Concentrate DX60. Big Gain Wisconsin, LLC. Lodi. Wisconsin), which supplied 39% crude protein, 1.2% crude fat, 3.7% calcium, and 0.50% phosphorus. The supplement also provided 69 mg of decoquinate/kg supplement for the prevention of coccidiosis.
